# Genetic Diversity of Intimin Gene of Atypical Enteropathogenic *Escherichia coli* Isolated from Human, Animals and Raw Meats in China

**DOI:** 10.1371/journal.pone.0152571

**Published:** 2016-03-31

**Authors:** Yanmei Xu, Xiangning Bai, Ailan Zhao, Wang Zhang, Pengbin Ba, Kai Liu, Yujuan Jin, Hong Wang, Qiusheng Guo, Hui Sun, Jianguo Xu, Yanwen Xiong

**Affiliations:** 1 State Key Laboratory for Infectious Disease Prevention and Control, Collaborative Innovation Center for Diagnosis and Treatment of Infectious Diseases, National Institute for Communicable Disease Control and Prevention, Chinese Center for Disease Control and Prevention, Beijing, China; 2 Longgang Center for Disease Control and Prevention, Shenzhen, Guangdong Province, China; 3 Zigong Center for Disease Control and Prevention, Zigong, Sichuan Province, China; 4 Suixian Center for Disease Control and Prevention, Shangqiu, Henan Province, China; University of Minnesota, UNITED STATES

## Abstract

Atypical enteropathogenic *Escherichia coli* (aEPEC) is considered to be an emerging enteropathogen that is more prevalent than typical EPEC in developing and developed countries. The major adherence factor, intimin, an outer membrane protein encoded by *eae*, plays a pivotal role in the pathogenesis of aEPEC. This study investigated the distribution and polymorphisms of intimin subtypes of 143 aEPEC strains from diarrheal patients, healthy carriers, animals, and raw meats in China. These aEPEC strains belonged to more than 71 different serotypes, which comprised 52 O serogroups and 24 H types. Sixty-eight different *eae* genotypes and 19 intimin subtypes were detected. Eighteen, eight, seven, and five intimin subtypes were identified from 86 diarrheal patients, 14 healthy carriers, 19 animals, and 24 raw meats strains, respectively. Intimin β1 was the most prevalent subtype in strains from diarrheal patients (34.88%) and animals (47.37%). There was a statistically significant difference in the distribution of *eae*-β1 between diarrheal patients and healthy carriers (*P* = 0.004). Intimin-θ was more predominant among raw meat strains (50%) than among diarrheal patients strains (12.79%, *P* = 0.0003), healthy carrier strains (7.14%, *P* = 0.007), or animal strains (15.79%, *P* = 0.020). The two predominant subtypes (*eae*-β1 and *eae*-θ) had considerable polymorphisms with no significant differences among the four sources. PFGE analysis revealed 119 distinct patterns and the strains were clustered into 11 groups with similarity indices ranging from 63% to 100%. These results suggest that in China, aEPEC strains from different sources are highly heterogeneous. Animals and raw meats are important sources of genetically diverse intimin-harboring aEPEC, which might serve as important transmission vehicles of these bacteria.

## Introduction

Enteropathogenic *Escherichia coli* (EPEC), the first pathotype of diarrheagenic *E*. *coli* (DEC) described, represents the main bacterial pathogen that causes severe diarrhea in China [1–3]. EPEC is divided into two pathotypes: typical EPEC (tEPEC), which contains an EPEC adherence factor (EAF), and atypical EPEC (aEPEC), which is devoid of EAF [4]. Atypical EPEC is considered to be an emerging enteropathogen [5, 6]. Recent data suggest that aEPEC is more prevalent than tEPEC in both developing and developed countries [7]. Additionally, epidemiological studies have shown an association between aEPEC and acute childhood diarrhea and diarrhea of prolonged duration [5, 8, 9]. The only reservoir of tEPEC is generally considered to be humans, whereas animals and humans can both be reservoirs of aEPEC [5, 8, 9]. In 1987, the World Health Organization recognized that EPEC comprises strains of 12 O serogroups: O26, O55, O86, O111, O114, O119, O125, O126, O127, O128, O142 and O158 [4]. However, the distribution and frequencies of serogroups and serotypes can vary considerably from region to region and over time [5, 8, 9].

EPEC has the ability to form attaching and effacing (A/E) lesions on intestinal epithelial cells [10]. The genes involved in the formation of A/E lesions are located in a chromosomal pathogenicity island called the locus of enterocyte effacement (LEE) [11]. The LEE encodes an outer membrane protein intimin that is encoded by the *eae* gene localized in the central region of the LEE. Intimin is an adherence factor that plays a pivotal role in intestinal colonization [12].

The intimin sequences are conserved in the N-terminal region, but highly variable in the last C-terminal region (280 amino acids), where cell binding activity is localized [4]. Analyses of the variable C-terminal encoding sequence of *eae* have revealed at least 30 distinct subtypes: α1, α2, α8, β1, β2, β3, γ1, γ2, ε1, ε2, ε3, ε4, ξ, ζ, ζ3, η, η2, θ, τ, ι1, ι2, κ, λ, μ, ν, υ, ο, π, ρ, and σ [13]. Researchers have postulated that intimin alleles influence host specificity and tissue tropism [14]. The association between different *eae* subtypes and particular *E*. *coli* serotypes or pathotypes has been used in the classification of strains from culture collections [15, 16]; however, tissue specificity and ecological associations between different *eae* subtypes in patients and healthy humans remain to be addressed. Moreover, minute variations (i.e., polymorphisms) within an *eae* subtype have rarely been described. Currently, the characteristics of aEPEC strains in China have not been well defined. In this study, we investigated the intimin subtypes and polymorphisms of aEPEC isolated from diarrheal patients, healthy carriers, animals, and raw meats in China.

## Materials and Methods

### Samples and isolation of aEPEC

A total of 3401 samples were collected from 2006 to 2014 by local centers for disease control and prevention. Among which, 1418 fecal samples were collected from diarrheal patients in sentinel hospitals, 640 fecal samples were collected from healthy humans during routine physical examination, 897 fecal samples of animals and 446 raw meat samples were collected in routine surveys, in six geographical regions in China (Beijing city, Guangdong province, Sichuan province, Henan province, Shanxi province, and Heilongjiang province). About 200 milligrams of each fecal sample or about one gram of each raw meat sample was enriched in 3 ml or 9 ml of modified Tryptone Soya Broth (mTSB) supplemented with novobiocin (10 μg/μl) (Oxoid, UK) and incubated at 37°C for 16 to 24 h with shaking at 200 rpm. Briefly, 1.5 ml of each enrichment sample was centrifuged and 150 μl of the lysis buffer (100 mM NaCl, 10 mM Tris–HCl [pH 8.3], 1 mM EDTA [pH 9.0], 1% Triton X-100) was used to suspend the centrifuged enrichment sample, boiled for 10 min and centrifuged. The supernatant was then used as template to detect the presence of *eae* gene by PCR assay [9]. The *eae*-positive enrichment culture was directly streaked onto CHROMagar^™^ ECC plate (CHROMagar, France) and incubated overnight at 37°C. On each plate, about ten *E*. *coli*-like colonies (blue or colorless) were picked randomly to test for the presence of *eae* gene. Then, the *eae*-positive colonies were plated onto Luria-Bertani (Oxoid, UK) plates and incubated overnight for further identification. All *eae*-positive *E*. *coli* isolates were confirmed by API 20E biochemical test strips (bioMérieux, France). Isolates with *eae*^+^, *stx*_1_^-^/*stx*_2_^-^, and *bfpA*^-^ were identified as aEPEC [9]. In total, 143 aEPEC strains were isolated from four different sources: diarrheal patients (n = 86), healthy carriers (n = 14), animals (cattle, pig, live chicken, bird; n = 19), and raw meats (beef, mutton, pork, chicken meat; n = 24) (Table 1 and S1 Table). Unless otherwise specified, bacteria were stored at -80°C and subcultured on Luria-Bertani agar at 37°C.

**Table 1:** Intimin subtypes of atypical EPEC strains isolated from diarrheal patients, healthy carriers, animals, and raw meats^a^.

Intimin subtype	Diarrheal patients	Healthy carriers	Animal	Raw meat	Subtotal
β1	30 (34.88)	0 (0)	9 (47.37)	4 (16.67)	43 (30.07)
θ	11 (12.79)	1 (7.14)	3 (15.79)	12 (50.00)	27 (18.88)
ε2	8 (9.30)	2 (14.28)	0 (0)	0 (0)	10 (6.99)
η2	5 (5.81)	3 (21.43)	0 (0)	0 (0)	8 (5.59)
κ	4 (4.65)	0 (0)	0 (0)	4 (16.67)	8 (5.59)
ζ3	2 (2.32)	3 (21.43)	1 (5.26)	2 (8.33)	8 (5.59)
ι1	6 (6.98)	1 (7.14)	0 (0)	0 (0)	7 (4.90)
ε1	0 (0)	1 (7.14)	2 (10.53)	2 (8.33)	5 (3.50)
ο	1 (1.16)	2 (14.28)	2 (10.53)	0 (0)	5 (3.50)
α1	2 (2.32)	1 (7.14)	1 (5.26)	0 (0)	4 (2.80)
γ1	2 (2.32)	0 (0)	1 (5.26)	0 (0)	3 (2.10)
λ	3 (3.49)	0 (0)	0 (0)	0 (0)	3 (2.10)
β2	2 (2.32)	0 (0)	0 (0)	0 (0)	2 (1.40)
μ	2 (2.32)	0 (0)	0 (0)	0 (0)	2 (1.40)
ι2	2 (2.32)	0 (0)	0 (0)	0 (0)	2 (1.40)
ξ	2 (2.32)	0 (0)	0 (0)	0 (0)	2 (1.40)
ζ	2 (2.32)	0 (0)	0 (0)	0 (0)	2 (1.40)
α2	1 (1.16)	0 (0)	0 (0)	0 (0)	1 (0.70)
π	1 (1.16)	0 (0)	0 (0)	0 (0)	1 (0.70)
**Total**	86 (100)	14 (100)	19 (100)	24 (100)	143 (100)

^a^ Values presented as number of strains (percentage).

### Serotyping of aEPEC strains

The O serogroups were screened by O-genotyping PCR created by Iguchi *et al*. [17]. The complete *E*. *coli* O antisera (Statens Serum Institute, Denmark) were used to confirm the PCR results. The H type of each isolate was determined by amplifying and sequencing the *fliC* gene with the primers fliC-F (5′-ATGGCACAAGTCATTAATACCCAAC-3′) and fliC-R (5′-CTAACCCTGCAGCAGAGACA-3′) reported by Fields *et al*. [18], and then comparing sequences in the SerotypeFinder database (https://cge.cbs.dtu.dk/services/SerotypeFinder/) [19].

### *eae* gene sequencing

Bacterial genomic DNA was prepared for PCR as previously described [13]. Subtyping of *eae* was performed by PCR amplification and sequencing a ~3.2-kb *cesT*-*escD* LEE region as reported by Ooka *et al*. with minor modifications [13]. Briefly, the 5′ half of the gene and its upstream region were amplified by PCR using the *cesT*-F9 (5′-TCAGGGAATAACATTAGAAA-3′)/*eae*-R3 (5′-TCTTGTGCGCTTTGGCTT-3′) primer pair, and the 3′ half and the downstream region were amplified using the *eae*-F1 (5′-ACTCCGATTCCTCTGGTGAC-3′)/*escD*-R1 (5′-GTATCAACATCTCCCGCCCA-3′) primer pair; the primer pairs generated ~1.6 and ~1.9 kb amplicons, respectively. PCR products were analyzed by agarose gel electrophoresis, purified with the QIAquick PCR purification kit (Qiagen, Germany), and sequenced using the ABI 3730 Automated DNA Analyzer (Applied Biosystems, USA). An additional primer *eae*-R3RC (5′-AAGCCAAAGCGCACAAGA-3′) was used for the complete sequencing of the 1.9 kb amplicon.

### *eae* gene subtyping and polymorphism analysis

The sequenced ~3.2-kb *cesT*-*escD* region was assembled with SeqMan II (DNASTAR Inc., USA). The reference *eae* sequences downloaded from GenBank [13] and the complete *eae* sequences obtained in this study were aligned using the ClustalW program, and genetic distances were calculated with MEGA 6 (www.megasoftware.net) [20]. A cutoff value of 95% nucleotide sequence identity was used to define a novel subtype as described previously [21]. A Neighbor-Joining tree based on the maximum composite likelihood model with 1,000 bootstrap resamplings was constructed with MEGA 6. All 143 complete *eae* sequences acquired in this study were submitted to GenBank with accession numbers KT591191–KT591333.

### Pulsed-field Gel Electrophoresis (PFGE)

PFGE was performed according to the PulseNet protocol developed for non-O157 *E*. *coli* (http://www.cdc.gov/pulsenet/PDF/ecoli-shigella-salmonella-pfge-protocol-508c.pdf). The genomic DNA was digested with 45 U of *Xba*I (Takara, China) at 37°C for 2 h. Macrorestriction fragments were resolved by counter-clamped homogeneous electric field electrophoresis in a CHEF-DRIII apparatus (Bio-Rad, USA). The run time was 19 h at 6.0 V/cm, with initial and final switch times of 6.76 s and 35.38 s, respectively. The image was captured with a Gel Doc XR+ software (Bio-Rad, USA). Data analysis was performed and an UPGMA dendrogram was constructed using Bionumerics (Version 4.0, Applied Maths BVBA, Belgium).

### Statistical analyses

The associations between isolate sources and *eae* subtypes or polymorphisms were evaluated by Chi-square or Fisher’s exact test. Statistical analyses were performed with Epi Info software [22]. *P* < 0.05 was considered statistically significant.

### Ethics statement

Fecal samples of humans were acquired with written informed consent of participants and animal fecal samples were collected with the consent of the owners of the lands and animals. Meat samples were purchased from markets. The study was approved by the ethics committee of National Institute for Communicable Disease Control and Prevention, China CDC, according to the medical research regulations of National Health and Family Planning Commission of the People's Republic of China.

## Results and Discussion

### Serotypes of aEPEC strains

The O-typeable aEPEC strains reported so far belong to >200 different serotypes [4]. Trabulsi *et al*. inferred that ten O serotypes: O26:H[11], O55:[H7], O55:H34, O86:H8, O111ac:[H8], O111:[H9], O111:H25, O119:H2, O125ac:H6, and O128:H2, were most frequently recognized worldwide [23]. In this study, more than 71 different serotypes were detected in the 143 aEPEC strains, which comprised 52 O serogroups and 24 H types (S1 Table). Thirteen isolates were O untypable because they did not react with any of the available O-typing sera (O1–O187) or could not be typed by the O-genotyping PCR method. Seven isolates were H untypable because they could not produce any PCR product. The most frequent serogroup was O51 (15/143, 10.48%), followed by O119 (12/143, 8.39%), O76 and O88 (7/143, 4.9% each). Approximately 80% of the 143 aEPEC strains did not belong to the classical EPEC serogroups and more than 90% of them did not belong to the most common aEPEC serotypes. The O51 serogroup comprised the most frequent serotype O51:H7 (10 isolates from diarrheal patients), and the O119 serogroup included the second frequent serotype O119:H21 (6 animal strains and 3 diarrheal patient strains). The strains from diarrheal patients, healthy carriers, animals and raw meats presented 49, 12, 12 and 14 serotypes, respectively (S1 Fig). Consequently, aEPEC serotypes in China showed vast diversity and difference from those in other countries [15, 24, 25].

### *eae* diversity in 143 aEPEC strains

The differentiation of intimin alleles represents an important tool for enterohemorrhagic *E*. *coli* (EHEC) and EPEC typing in routine diagnostics, pathogenesis, and epidemiological, clonal, and immunological studies. Most studies have focused on the allele distribution between diarrhea and control samples, or have characterized the intimin subtypes of EPEC strains from animal and human sources [21, 24–26]. This study characterized the distribution of intimin subtypes of aEPEC strains isolated not only from diarrheal patients, healthy carriers, and animals, but also from raw meat samples, and analyzed the polymorphisms of the predominant intimin subtypes to understand their role in human diarrhea.

**Fig 1:**
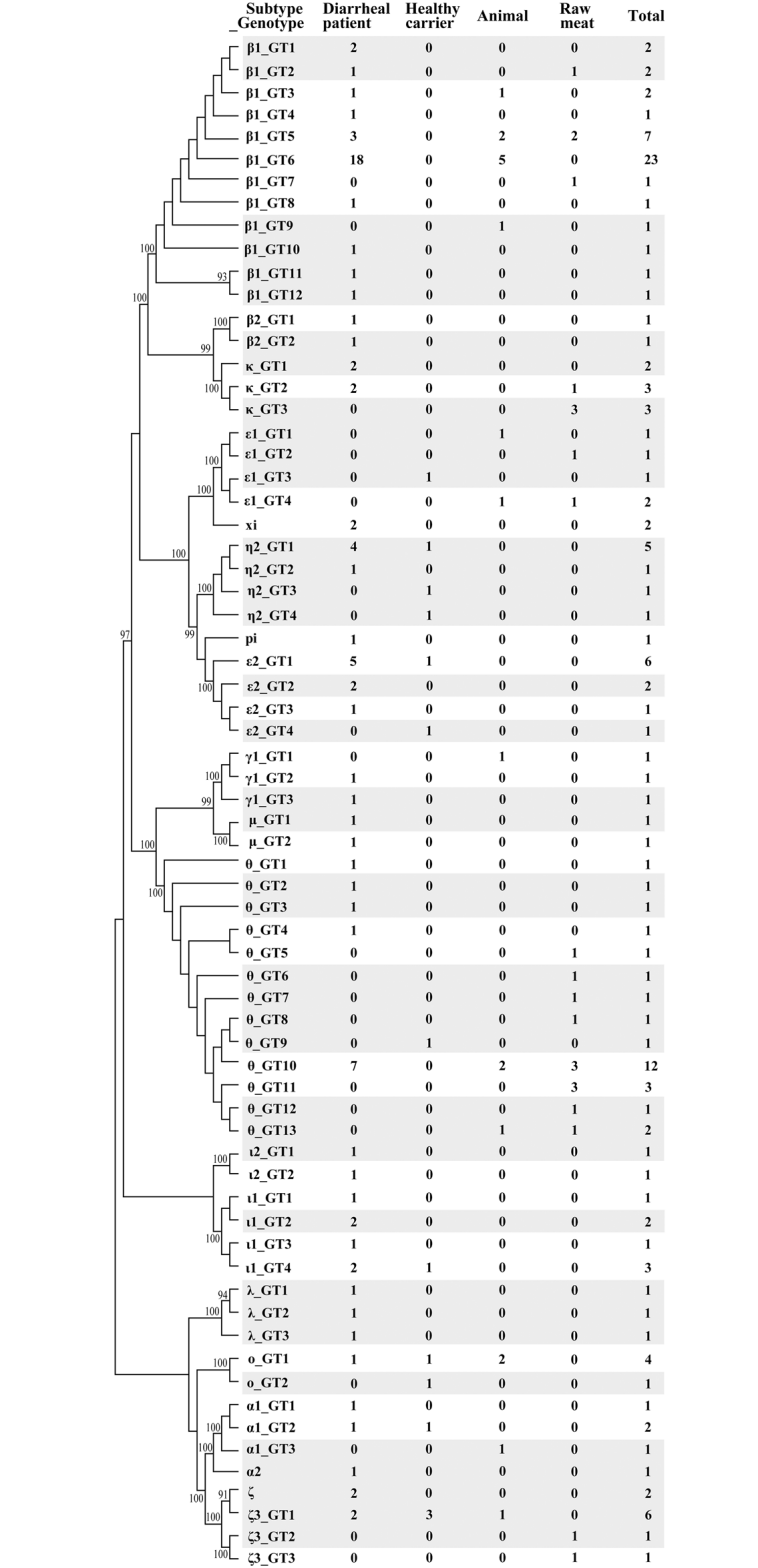
Neighbor-joining tree of 143 aEPEC strains analyzed by *eae* genes. The corresponding subtypes, genotypes (abbreviated as GT), and number of strains isolated from different sources are listed. The sequences of 39 *eae* genotypes firstly identified in this study were shadowed.

Based on pairwise comparisons, the *eae* sequences of the 143 aEPEC strains varied from each other in 0–19.30% of nucleotide sites. Therefore, there was 80.70–100% identity among the sequences of the 143 aEPEC strains. There were 68 different *eae* sequence types (genotypes) in the 143 aEPEC strains. Except for two major genotypes containing 23 and 12 strains each, 44 genotypes contained only one strain isolated from one of the four sources, while the rest contained two to seven strains (Fig 1). Notably, eight genotypes (β1_GT2, β1_GT3, β1_GT5, β1_GT6, κ_GT2, θ_GT10, ο_GT1 and ζ3_GT1) were simultaneously present in diarrheal patient strains and animal/raw meat strains, which revealed that animals and raw meats could serve as important transmission vehicles of these bacteria. Six genotypes (η2_GT1, ε2_GT1, ι1_GT4, ο_GT1, α1_GT2 and ζ3_GT1) contained strains isolated from diarrheal and healthy humans. While, based on the phylogenetic analyses of *eae* genes at the amino acid level, 59 different types were produced. It indicated that synonymous nucleotide changes occurred in nine of 68 *eae* genotypes. The BLAST analysis of the 143 *eae* sequences revealed that 58 sequences belonging to 39 *eae* genotypes (indicated by grey shadow in Fig 1) were distinct from the sequences in the GenBank, while 85 sequences belonging to 29 *eae* genotypes were identical to the sequences in the database. These data suggested that most *eae* genotypes (39/68) of aEPEC strains from China had not been identified previously.

### *eae* subtypes among 143 aEPEC strains

The 143 aEPEC strains had a wide variety of intimin subtypes. Overall, there were 19 intimin variants: β1 (43 strains), θ (27 strains), ε2 (10 strains), κ (eight strains), η2 (eight strains), ζ3 (eight strains), ι1 (seven strains), ο (five strains), ε1 (five strains), α1 (four strains), λ (three strains), γ1 (three strains), ι2 (two strains), ξ (two strains), μ (two strains), β2 (two strains), ζ (two strains), α2 (one strain), and π (one strain). None of the strains had intimin subtypes α8, β3, ε3, ε4, η, γ2, ν, ρ, τ, υ, or σ (Table 1).

The strains isolated from diarrheal patients had more intimin types than the strains isolated from healthy carriers, animals, or raw meats. Almost all of the 19 intimin subtypes (except for intimin subtype ε1) were detected in 86 diarrheal patients, but only eight, seven, and five intimin subtypes were identified in strains from healthy carriers, animals, and raw meats, respectively. Previously, intimin α2, η, and μ have been mainly detected in human EPEC strains [27–30], with the exception of one intimin α2 strain from cat [30] and one intimin η2 strain from cattle [31]. In our study, eight *eae* subtypes were only detected in diarrheal patients, including intimin subtypes λ, β2, μ, ι2, ξ, ζ, α2, and π. Intimin γ1 was only detected in diarrheal patients and pig. Intimin κ was only identified in diarrheal patients and raw meats, while *eae*-ε2, η2, and ι1 were only present in humans (including diarrheal patients and healthy carriers). There were significant differences in the overall distribution of the intimin subtypes among diarrheal patients, healthy carriers, animals, and raw meats (*P* = 0.0034). In this study, the distribution of intimin alleles was similar to that reported by other researchers; however, the order of frequency was different [25, 32].

The *eae*-β and *eae*-γ subtypes are considered to be two most frequent variants in animal isolates and clinical isolates associated with human diarrheal diseases [21, 25]. Zhang *et al*. reported that *eae*-β and *eae*-γ are present in 34.2 and 31.5%, respectively, of EPEC strains from diarrheal patients in Germany [33], while Blanco *et al*. reported that *eae*-β and *eae*-γ are present in 28.6 and 38.6%, respectively, of EHEC isolates from diarrheal patients in Spain [31]. In our study, *eae*-β1 was the most prevalent intimin subtype containing 43 (30.07%) aEPEC strains. Additionally, *eae*-β1 was the most predominant intimin subtype in strains from diarrheal patients (30/86, 34.88%) and animals (9/19, 47.37%). However, none of the strains from healthy carriers contained *eae*-β1. There was a statistically significant difference in the distribution of *eae*-β1 among the four sources (*P* = 0.0084). The distribution of *eae*-β1 in diarrheal patients was significantly higher than that in healthy carriers (*P* = 0.004), similar to the findings of Wang *et al*.[15].

The second most prevalent allele was *eae*-θ (18.88%), which was identified in 11 strains from diarrheal patients, 12 strains from raw meats, three strains from animals, and one strain from healthy carriers. Notably, half of the strains from raw meats harbored the *eae*-θ allele. The overall prevalence of *eae*-θ in human, animal, and raw meat isolates was significant (*P* = 0.0003). Intimin θ was more predominant among the raw meat strains than among the diarrheal patient strains (12.79%, *P* = 0.0003), healthy carrier strains (7.14%, *P* = 0.007), or animal strains (15.79%, *P* = 0.020). The higher frequency of *eae*-θ has been reported in children with diarrhea [34] and animals [35]. The results of this study revealed a possible risk for humans due to the high prevalence of the *eae*-θ subtype in raw meats. Raw meats represent a possible transmission route of this emerging pathogen.

The *eae*-γ subtype represents one of the most frequent variants in animal isolates and clinical isolates associated with human diarrheal diseases and is more frequently isolated in diarrhea cases of longer duration (>7 d) than those of shorter duration [24]. Our results showed that only three (2.10%) aEPEC strains (one O145:H28 from animal, one O157:H7 and one O55:H7 from diarrheal patients) harbored *eae*-γ1. These three isolates belonged to serotypes more commonly associated with Shiga toxin-producing *E*. *coli* (STEC) /enterohemorrhagic *E*. *coli* (EHEC) strains [10]. The results support the conclusion that aEPEC strains appear to be that STEC have lost the Shiga toxin encoding bacteriophage(s) during passage through the intestine [36]. The low prevalence of *eae*-γ in aEPEC could be attributed to the fact that *eae*-γ is commonly associated with *stx*-positive EHEC strains such as O157:H7 and O55:H7 that were not examined in this study [37].

In this study, *eae*-κ was present in eight (5.59%) aEPEC strains (O118/O88:H5 and O157:H39/NT from four diarrheal patients, and O37/O49/O61:H10 from four raw meats). It has been reported that *eae*-κ is more common in animal feces and that it is associated with more severe episodes of diarrhea in humans in comparison with other alleles [24]. Raw meats may represent a possible transmission route of *eae*-κ strains.

*eae*-ζ3 strains were obtained in aEPEC isolates from all of four sources with non-significant differences in the prevalence rates. Few studies have reported that aEPEC contained intimin α1, α2, β2, γ1, γ3, λ, ι2, and ε8. *eae*-α1, *eae*-ξR/β2, and *eae*-γ1 strains have been isolated from children with diarrhea [34], *eae*-ι2 strains have been isolated from cattle [38], and *eae*-λ strains have been isolated from children [39] in Uruguay, New Zealand and India, respectively. Intimin α1, α2, β2, γ1, ξ, ν, λ, π, and ι2 were not identified in humans, animals, or raw meat sources in this study, possibly due to the limitation of strains or low prevalence rates.

### Polymorphisms in *eae* subtypes

**Fig 2:**
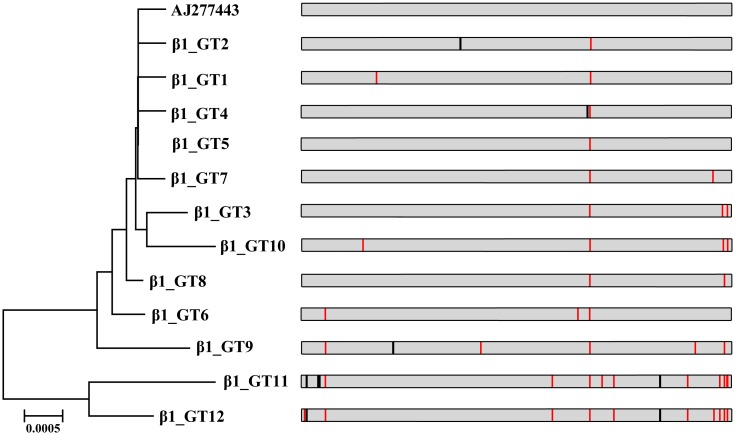
Neighbor-joining tree of 12 genotypes of *eae* genes among the 43 *eae*-β1 strains and one reference sequence (AJ277443). Polymorphic sites in the intimin alleles were illustrated. Black vertical lines indicate synonymous polymorphic sites and red vertical lines represent non-synonymous polymorphic sites. GT: genotype.

The polymorphisms of *eae*-β1 and *eae*-θ, which were the most prevalent subtypes in this study, were analyzed. Among the 43 *eae*-β1 strains, 26 polymorphisms were detected based on the *eae*-β1 reference sequence (GenBank accession number AJ277443) and 12 genotypes were defined (Fig 2). Out of seven synonymous polymorphisms, three were localized in the periplasmic (PP) domain, two were situated in the transmembrane (TM) domain, and two were present in the extracellular (EC) domain. Fourteen out of 19 non-synonymous polymorphisms were positioned in the EC domain of 3′-regions of the *eae* gene. The results were consistent with the observation that the 3′-regions of *eae* genes are highly heterogeneous [34]. The highly variable C-terminal extracellular domains, where the active receptor-binding site resides [40], are responsible for receptor binding to epithelial cells. Studies have reported that intimin alleles influence host specificity and tissue tropism [41]. Two major genotypes, GT6 and GT5, were detected in 23 strains (18 belonged to O51:H7, O88:H25, O119/O21/O156:H21 from diarrheal patients and five belonged to O103/O119:H21 from animals) and seven strains (three belonged to O26:H11, O111:H9, O128:H2 from diarrheal patients, two belonged to O26:H11, O71:H11 from raw meats, and two belonged to O26:H11 from animals), respectively. The rest of the genotypes were represented by only one or two strains. However, at the amino acid level, GT2 strains (one from diarrheal patient and one from mutton) and GT4 strain (one from diarrheal patient) possessed identical sequence to the seven strains of GT5, because of the presence of synonymous nucleotide changes. The most variable genotype was GT11 containing one O45:H11 strain isolated from diarrheal patient, which contained 14 polymorphisms (including four synonymous polymorphisms and ten non-synonymous polymorphisms). Six *eae*-β1 genotypes (β1_GT1, β1_GT2, β1_GT9-12) were novel compared to publicly available database (GenBank) by BLAST analysis. In contrast, Marjorie *et al*. reported that *eae*-β1 polymorphisms were uncommon or predominantly synonymous [42]. However, there was no statistically significant differences in the presence of polymorphisms among the four sample sources from this study. Even though the *eae*-β1 subtype was the predominant intimin variant in clinical isolates, it has been demonstrated that the invasion of differentiated intestinal Caco-2 cell is a sporadic property among aEPEC strains carrying common intimin subtypes [43]. Therefore, only some of the β1 genotypes/polymorphisms with certain virulence profiles may facilitate aEPEC pathogenesis. Further studies are required to determine the roles of specific genotypes/polymorphisms in the clinical features of the disease.

**Fig 3:**
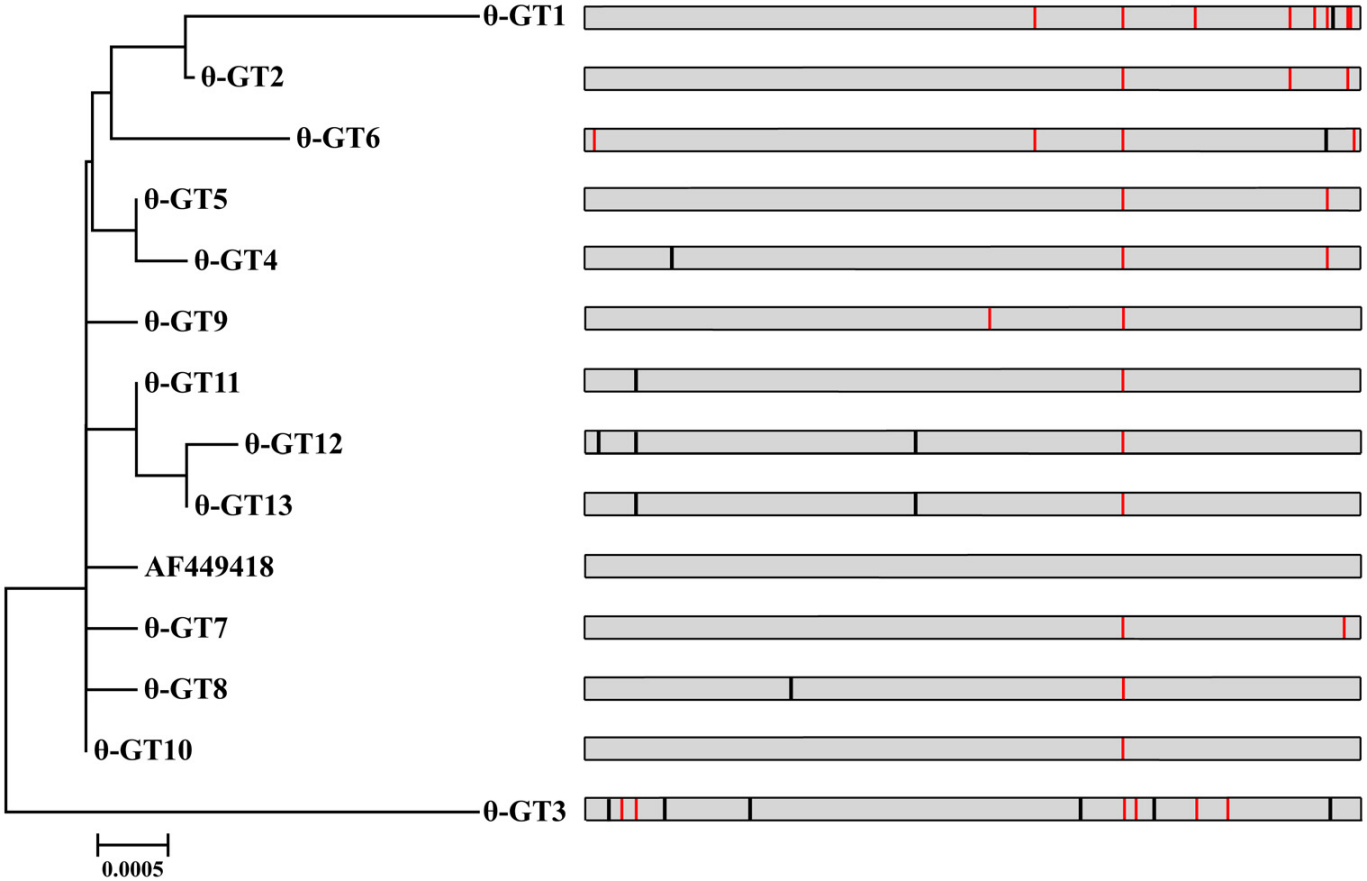
Neighbor-joining tree of 13 genotypes of *eae* genes among the 27 *eae*-θ strains and one reference sequence (AF449418). Polymorphic sites in the intimin alleles were illustrated. Black vertical lines indicate synonymous polymorphic sites and red vertical lines represent non-synonymous polymorphic sites. GT: genotype.

Thirty polymorphisms were detected in the second most prevalent subtype (*eae*-θ) based on the *eae*-θ reference sequence (GenBank accession number AF449418); 13 genotypes were defined. Seventeen polymorphisms were non-synonymous, while 13 were synonymous (Fig 3). Eight and five of the 13 genotypes were detected in raw meat and diarrheal patient isolates, respectively. The most prevalent genotype, GT10, contained 12 isolates (seven belonged to O51/O136:H40, ONT:H16 from diarrheal patients, three belonged to O2:H40, O156:H8 from raw meats, and two belonged to O119:H21 from animals). However, at the amino acid level, the GT8 strain (O2:H40 from chicken meat), the three GT11 strains (O76:H7 from raw meats), the GT12 strain (O76:H7 from raw meat) and the two GT13 strains (O76:H7 from raw meat and animal) possessed identical sequence to the 12 strains of GT10, due to the occurrence of synonymous nucleotide changes. Consequently, a total of 19 strains had identical intimin amino acid sequence. No statistically significant differences were observed among the four sources. To the best of our knowledge, this is the first study that reports the polymorphisms of *eae*-θ.

### Genetic diversity of the aEPEC strains by PFGE analysis

DNA macrorestriction analysis by PFGE has been considered the ‘gold standard’ for the molecular subtyping of many pathogenic organisms, including EPEC strains [34]. The overall heterogeneity of aEPEC in this study was also revealed by PFGE analysis, showing that the 143 aEPEC strains (five of them failed to produce distinguishable bands) investigated in our study belonged to 119 distinct pattern types (PTs) with similarity indices ranging from 63% to 100%, considering a difference of at least one restriction fragment in the patterns as the criterion for discriminating between them (S1 Fig). In the dendrogram produced by the UPGMA algorithm, few strains showed identical PT and the strains were clustered into 11 groups within 67% similarity according to the Dice similarity index. The predominant group III contained 75 isolates. All of the O51:H7 strains containing intimin-β1, O119:H21 strains containing intimin-θ or β1 and O76:H7 strains containing intimin-θ from diarrheal patients, animals or raw meats were clustered in group III. Five strains belonged to O119:H21/H25 and intimin-θ or β1 isolated from different sources (two from *Egretta garzetta*, two from diarrheal patients and one from mutton) showed identical PT. Four strains with identical PT showed the same serotype (O51:H7), intimin subtype (β1) and source (diarrheal patients). It has been found that the pathogenesis of aEPEC seemed to be related to the serotypes [15]. aEPEC strains with several serotypes such as O127a:K63 [44], O39:NM [45], O55:HNM [46], and O76 [47] have been reported to cause outbreaks linked to diarrhea. In our study, aEPEC strains with O51:H7 and *eae*-β1 clustered in group III seemed to be specific among diarrheal patients. But the large variety of serotypes, phylogenetic properties and intimin subtypes present in both healthy and diseased human isolates makes it difficult to determine which strains are truly high pathogenic.

In conclusion, aEPEC strains isolated from diarrheal patients, healthy carriers, animal feces, and raw meat samples in China were highly heterogeneous in terms of O:H serotypes, PFGE patterns and intimin subtypes. Even though the main intimin subtype identified in strains isolated from human clinical and animal feces was *eae*-β1 and the predominant subtype detected in raw meat-derived strains was *eae*-θ, isolates from diarrheal patients and animal/raw meats harbored identical serotypes, PFGE patterns or *eae*-subtypes and even *eae* genotypes. These results suggested that animals and raw meats are reservoirs of aEPEC possessing these properties in China and represent disease transmission vehicles.

## Supporting Information

S1 Fig PFGE profiles of the 138 aEPEC strains investigated in the study. The dendrogram was generated by Bionumeric software. The strains were clustered into 11 groups generated by the UPGMA algorithm of 67% similarity according to the Dice index.
(TIF)

S1 Table Intimin subtypes and GenBank accession numbers of the 143 aEPEC strains used in this study. (DOCX)
