# Supplementary figures and images for: Genetic Diversity of Intimin Gene of Atypical Enteropathogenic Escherichia coli Isolated from Human, Animals and Raw Meats in China
Source: PLoS One. 2016 Mar 31;11(3):e0152571. doi: 10.1371/journal.pone.0152571 (PMC4816571; doi:10.1371/journal.pone.0152571)

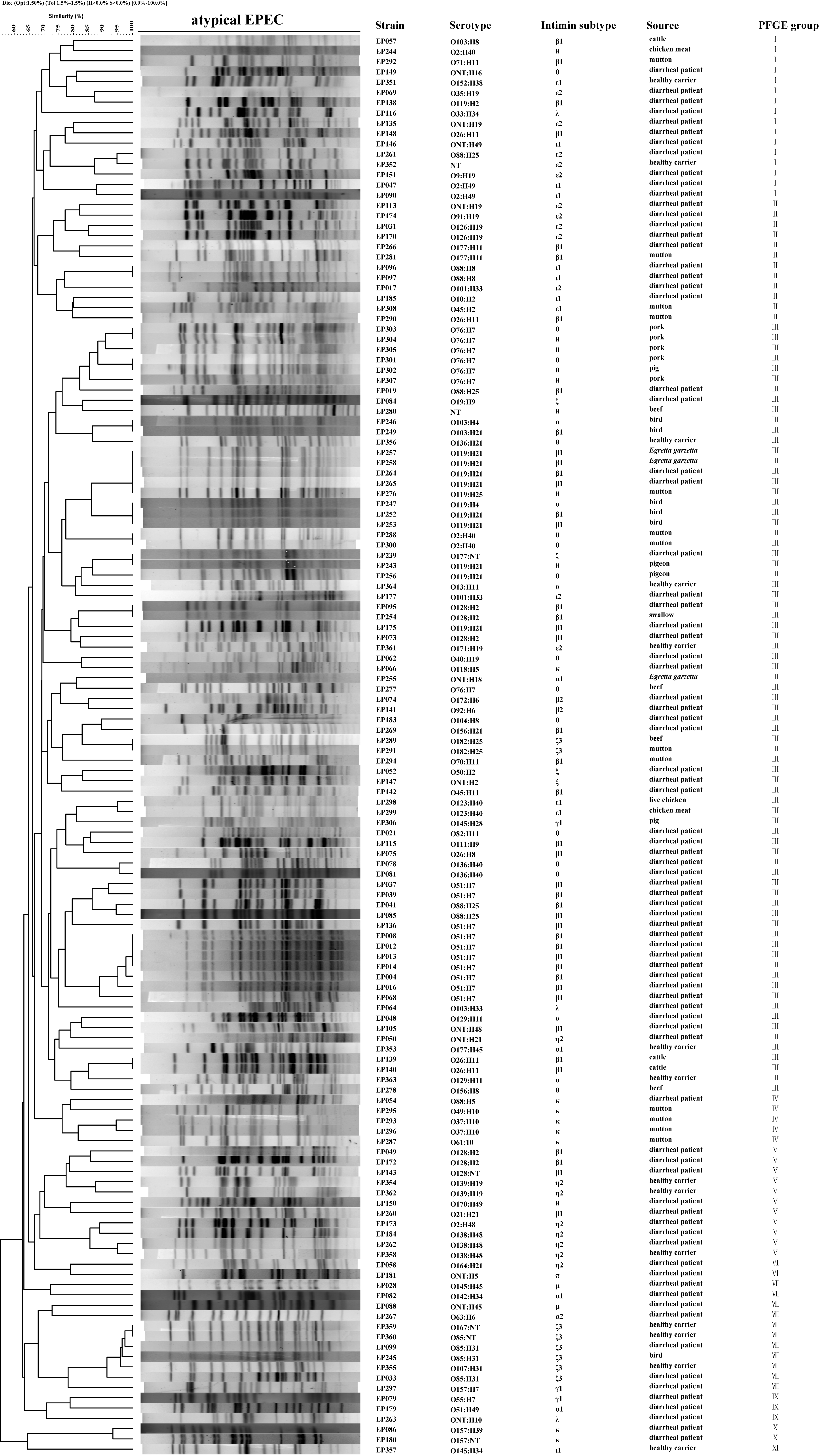

Supplement: S1 Fig — The dendrogram was generated by Bionumeric software. The strains were clustered into 11 groups generated by the UPGMA algorithm of 67% similarity according to the Dice index. (TIF) [file pone.0152571.s001.tif]
