# Supplementary material for: Genetic Diversity of Intimin Gene of Atypical Enteropathogenic Escherichia coli Isolated from Human, Animals and Raw Meats in China
Source: PLoS One. 2016 Mar 31;11(3):e0152571. doi: 10.1371/journal.pone.0152571 (PMC4816571; doi:10.1371/journal.pone.0152571)
Supplement: S1 Table — (DOCX) [file pone.0152571.s002.docx]

**Table S1. Intimin subtypes and GenBank accession numbers of the 143 aEPEC strains used in this study**

| **Strain** | **Source** | **Isolation Year** | **Location (province)** | **Serotype ^a^** | **Intimin subtype** | **Allele size (bp)** | **GenBank accession No.** |
| --- | --- | --- | --- | --- | --- | --- | --- |
| EP004 | diarrheal patient | 2011 | Henan | O51:H7 | β1 | 2820 | KT591196 |
| EP008 | diarrheall patient | 2011 | Henan | O51:H7 | β1 | 2820 | KT591195 |
| EP012 | diarrheall patient | 2011 | Henan | O51:H7 | β1 | 2820 | KT591194 |
| EP013 | diarrheal patient | 2011 | Henan | O51:H7 | β1 | 2820 | KT591193 |
| EP014 | diarrheal patient | 2011 | Henan | O51:H7 | β1 | 2820 | KT591192 |
| EP016 | diarrheal patient | 2011 | Henan | O51:H7 | β1 | 2820 | KT591191 |
| EP017 | diarrheal patient | 2011 | Henan | O101:H33 | ι2 | 2814 | KT591297 |
| EP019 | diarrheal patient | 2011 | Henan | O88:H25 | β1 | 2820 | KT591233 |
| EP021 | diarrheal patient | 2011 | Henan | O82:H11 | θ | 2808 | KT591240 |
| EP028 | diarrheal patient | 2011 | Henan | O145:H45 | μ | 2808 | KT591316 |
| EP031 | diarrheal patient | 2011 | Henan | O126:H19 | ε2 | 2847 | KT591274 |
| EP033 | diarrheal patient | 2011 | Henan | O85:H31 | ζ3 | 2817 | KT591323 |
| EP035 | diarrheal patient | 2011 | Henan | O51:H40 | θ | 2808 | KT591239 |
| EP037 | diarrheal patient | 2011 | Henan | O51:H7 | β1 | 2820 | KT591232 |
| EP039 | diarrheal patient | 2011 | Henan | O51:H7 | β1 | 2820 | KT591231 |
| EP041 | diarrheal patient | 2011 | Henan | O88:H25 | β1 | 2820 | KT591230 |
| EP047 | diarrheal patient | 2011 | Henan | O2:H49 | ι1 | 2814 | KT591296 |
| EP048 | diarrheal patient | 2010 | Shanxi | O129:H11 | ο | 2820 | KT591315 |
| EP049 | diarrheal patient | 2010 | Shanxi | O128:H2 | β1 | 2820 | KT591229 |
| EP050 | diarrheal patient | 2010 | Shanxi | ONT:H21 | η2 | 2847 | KT591288 |
| EP052 | diarrheal patient | 2006 | Shanxi | O50:H2 | ξ | 2847 | KT591314 |
| EP054 | diarrheal patient | 2006 | Shanxi | O88:H5 | κ | 2820 | KT591307 |
| EP057 | cattle | 2009 | Heilongjiang | O103:H8 | β1 | 2820 | KT591228 |
| EP058 | diarrheal patient | 2011 | Beijing | O164:H21 | η2 | 2847 | KT591287 |
| EP062 | diarrheal patient | 2011 | Henan | O40:H19 | θ | 2808 | KT591238 |
| EP064 | diarrheal patient | 2011 | Henan | O103:H33 | λ | 2817 | KT591262 |
| EP066 | diarrheal patient | 2011 | Henan | O118:H5 | κ | 2820 | KT591306 |
| EP068 | diarrheal patient | 2011 | Henan | O51:H7 | β1 | 2820 | KT591227 |
| EP069 | diarrheal patient | 2011 | Henan | O35:H19 | ε2 | 2847 | KT591273 |
| EP073 | diarrheal patient | 2011 | Henan | O128:H2 | β1 | 2820 | KT591226 |
| EP074 | diarrheal patient | 2011 | Henan | O172:H6 | β2 | 2820 | KT591333 |
| EP075 | diarrheal patient | 2011 | Henan | O26:H8 | β1 | 2820 | KT591225 |
| EP078 | diarrheal patient | 2011 | Henan | O136:H40 | θ | 2808 | KT591237 |
| EP079 | diarrheal patient | 2011 | Henan | O55:H7 | γ1 | 2805 | KT591261 |
| EP081 | diarrheal patient | 2011 | Henan | O136:H40 | θ | 2808 | KT591236 |
| EP082 | diarrheal patient | 2011 | Henan | O142:H34 | α1 | 2820 | KT591269 |
| EP084 | diarrheal patient | 2011 | Henan | O19:H9 | ζ | 2817 | KT591322 |
| EP085 | diarrheal patient | 2011 | Henan | O88:H25 | β1 | 2820 | KT591224 |
| EP086 | diarrheal patient | 2011 | Henan | O157:H39 | κ | 2820 | KT591305 |
| EP088 | diarrheal patient | 2011 | Henan | ONT:H45 | μ | 2808 | KT591313 |
| EP090 | diarrheal patient | 2011 | Henan | O2:H49 | ι1 | 2814 | KT591295 |
| EP091 | diarrheal patient | 2011 | Henan | O51:H40 | θ | 2808 | KT591235 |
| EP093 | diarrheal patient | 2011 | Henan | O51:H40 | θ | 2808 | KT591234 |
| EP095 | diarrheal patient | 2011 | Henan | O128:H2 | β1 | 2820 | KT591223 |
| EP096 | diarrheal patient | 2011 | Henan | O88:H8 | ι1 | 2814 | KT591303 |
| EP097 | diarrheal patient | 2011 | Henan | O88:H8 | ι1 | 2814 | KT591302 |
| EP099 | diarrheal patient | 2011 | Henan | O85:H31 | ζ3 | 2817 | KT591331 |
| EP105 | diarrheal patient | 2012 | Henan | ONT:H48 | β1 | 2820 | KT591222 |
| EP113 | diarrheal patient | 2012 | Henan | ONT:H19 | ε2 | 2847 | KT591272 |
| EP115 | diarrheal patient | 2012 | Henan | O111:H9 | β1 | 2820 | KT591221 |
| EP116 | diarrheal patient | 2012 | Henan | O33:H34 | λ | 2817 | KT591266 |
| EP118 | diarrheal patient | 2011 | Henan | O51:H40 | θ | 2808 | KT591260 |
| EP135 | diarrheal patient | 2010 | Shanxi | ONT:H19 | ε2 | 2847 | KT591286 |
| EP136 | diarrheal patient | 2010 | Shanxi | O51:H7 | β1 | 2820 | KT591220 |
| EP138 | diarrheal patient | 2011 | Beijing | O119:H2 | β1 | 2820 | KT591219 |
| EP139 | cattle | 2012 | Heilongjiang | O26:H11 | β1 | 2820 | KT591218 |
| EP140 | cattle | 2012 | Heilongjiang | O26:H11 | β1 | 2820 | KT591217 |
| EP141 | diarrheal patient | 2010 | Guangdong | O92:H6 | β2 | 2820 | KT591332 |
| EP142 | diarrheal patient | 2012 | Henan | O45:H11 | β1 | 2820 | KT591216 |
| EP143 | diarrheal patient | 2012 | Henan | O128:NT | β1 | 2820 | KT591215 |
| EP146 | diarrheal patient | 2012 | Beijing | ONT:H49 | ι1 | 2814 | KT591301 |
| EP147 | diarrheal patient | 2012 | Beijing | ONT:H2 | ξ | 2847 | KT591312 |
| EP148 | diarrheal patient | 2012 | Beijing | O26:H11 | β1 | 2820 | KT591214 |
| EP149 | diarrheal patient | 2012 | Beijing | ONT:H16 | θ | 2808 | KT591259 |
| EP150 | diarrheal patient | 2012 | Beijing | O170:H49 | θ | 2808 | KT591258 |
| EP151 | diarrheal patient | 2012 | Beijing | O9:H19 | ε2 | 2847 | KT591285 |
| EP170 | diarrheal patient | 2012 | Shanxi | O126:H19 | ε2 | 2847 | KT591284 |
| EP172 | diarrheal patient | 2012 | Shanxi | O128:H2 | β1 | 2820 | KT591213 |
| EP173 | diarrheal patient | 2012 | Shanxi | O2:H48 | η2 | 2847 | KT591294 |
| EP174 | diarrheal patient | 2012 | Shanxi | O91:H19 | ε2 | 2847 | KT591283 |
| EP175 | diarrheal patient | 2012 | Shanxi | O119:H21 | β1 | 2820 | KT591212 |
| EP177 | diarrheal patient | 2012 | Shanxi | O101:H33 | ι2 | 2814 | KT591300 |
| EP179 | diarrheal patient | 2012 | Shanxi | O51:H49 | α1 | 2820 | KT591268 |
| EP180 | diarrheal patient | 2012 | Shanxi | O157:NT | κ | 2820 | KT591304 |
| EP181 | diarrheal patient | 2012 | Henan | ONT:H5 | π | 2847 | KT591321 |
| EP183 | diarrheal patient | 2013 | Henan | O104:H8 | θ | 2808 | KT591257 |
| EP184 | diarrheal patient | 2013 | Henan | O138:H48 | η2 | 2847 | KT591293 |
| EP185 | diarrheal patient | 2013 | Henan | O10:H2 | ι1 | 2814 | KT591299 |
| EP239 | diarrheal patient | 2013 | Guangdong | O177:NT | ζ | 2817 | KT591330 |
| EP243 | pigeon | 2013 | Sichuan | O119:H21 | θ | 2808 | KT591256 |
| EP244 | chicken meat | 2013 | Sichuan | O2:H40 | θ | 2808 | KT591255 |
| EP245 | bird | 2013 | Sichuan | O85:H31 | ζ3 | 2817 | KT591329 |
| EP246 | bird | 2013 | Sichuan | O103:H4 | ο | 2820 | KT591320 |
| EP247 | bird | 2013 | Sichuan | O119:H4 | ο | 2820 | KT591319 |
| EP249 | bird | 2013 | Sichuan | O103:H21 | β1 | 2820 | KT591211 |
| EP252 | bird | 2013 | Sichuan | O119:H21 | β1 | 2820 | KT591210 |
| EP253 | bird | 2013 | Sichuan | O119:H21 | β1 | 2820 | KT591209 |
| EP254 | Swallow | 2013 | Sichuan | O128:H2 | β1 | 2820 | KT591208 |
| EP255 | Egretta garzetta | 2013 | Sichuan | ONT:H18 | α1 | 2820 | KT591267 |
| EP256 | pigeon | 2013 | Sichuan | O119:H21 | θ | 2808 | KT591254 |
| EP257 | Egretta garzetta | 2013 | Sichuan | O119:H21 | β1 | 2820 | KT591207 |
| EP258 | Egretta garzetta | 2013 | Sichuan | O119:H21 | β1 | 2820 | KT591206 |
| EP259 | Swallow | 2013 | Sichuan | O157:NT | ε1 | 2847 | KT591282 |
| EP260 | diarrheal patient | 2013 | Sichuan | O21:H21 | β1 | 2820 | KT591205 |
| EP261 | diarrheal patient | 2013 | Sichuan | O88:H25 | ε2 | 2847 | KT591281 |
| EP262 | diarrheal patient | 2013 | Sichuan | O138:H48 | η2 | 2847 | KT591292 |
| EP263 | diarrheal patient | 2013 | Sichuan | ONT:H10 | λ | 2817 | KT591265 |
| EP264 | diarrheal patient | 2013 | Sichuan | O119:H21 | β1 | 2820 | KT591204 |
| EP265 | diarrheal patient | 2013 | Sichuan | O119:H21 | β1 | 2820 | KT591203 |
| EP266 | diarrheal patient | 2013 | Sichuan | O177:H11 | β1 | 2820 | KT591202 |
| EP267 | diarrheal patient | 2013 | Sichuan | O63:H6 | α2 | 2820 | KT591271 |
| EP269 | diarrheal patient | 2013 | Sichuan | O156:H21 | β1 | 2820 | KT591201 |
| EP276 | mutton | 2014 | Beijing | O119:H25 | θ | 2808 | KT591253 |
| EP277 | beef | 2014 | Beijing | O76:H7 | θ | 2808 | KT591252 |
| EP278 | beef | 2014 | Beijing | O156:H8 | θ | 2808 | KT591251 |
| EP280 | beef | 2014 | Beijing | NT | θ | 2808 | KT591250 |
| EP281 | mutton | 2014 | Beijing | O177:H11 | β1 | 2820 | KT591200 |
| EP287 | mutton | 2014 | Beijing | O61:H10 | κ | 2820 | KT591311 |
| EP288 | mutton | 2014 | Beijing | O2:H40 | θ | 2808 | KT591249 |
| EP289 | beef | 2014 | Beijing | O182:H25 | ζ3 | 2817 | KT591328 |
| EP290 | mutton | 2014 | Beijing | O26:H11 | β1 | 2820 | KT591199 |
| EP291 | mutton | 2014 | Beijing | O182:H25 | ζ3 | 2817 | KT591327 |
| EP292 | mutton | 2014 | Beijing | O71:H11 | β1 | 2820 | KT591198 |
| EP293 | mutton | 2014 | Beijing | O37:H10 | κ | 2820 | KT591310 |
| EP294 | mutton | 2014 | Beijing | O70:H11 | β1 | 2820 | KT591197 |
| EP295 | mutton | 2014 | Beijing | O49:H10 | κ | 2820 | KT591309 |
| EP296 | mutton | 2014 | Beijing | O37:H10 | κ | 2820 | KT591308 |
| EP297 | diarrheal patient | unknown | Henan | O157:NT | γ1 | 2805 | KT591264 |
| EP298 | live chicken | 2014 | Sichuan | O123:H40 | ε1 | 2847 | KT591280 |
| EP299 | chicken meat | 2014 | Sichuan | O123:H40 | ε1 | 2847 | KT591279 |
| EP300 | mutton | 2014 | Beijing | O2:H40 | θ | 2808 | KT591248 |
| EP301 | pork | 2014 | Beijing | O76:H7 | θ | 2808 | KT591247 |
| EP302 | pig | 2014 | Beijing | O76:H7 | θ | 2808 | KT591246 |
| EP303 | pork | 2014 | Beijing | O76:H7 | θ | 2808 | KT591245 |
| EP304 | pork | 2014 | Beijing | O76:H7 | θ | 2808 | KT591244 |
| EP305 | pork | 2014 | Beijing | O76:H7 | θ | 2808 | KT591243 |
| EP306 | pig | 2014 | Beijing | O145:H28 | γ1 | 2805 | KT591263 |
| EP307 | pork | 2014 | Beijing | O76:H7 | θ | 2808 | KT591242 |
| EP308 | mutton | 2014 | Beijing | O45:H2 | ε1 | 2847 | KT591278 |
| EP351 | healthy carrier | 2014 | Guangdong | O152:H38 | ε1 | 2847 | KT591277 |
| EP352 | healthy carrier | 2014 | Guangdong | NT | ε2 | 2847 | KT591276 |
| EP353 | healthy carrier | 2014 | Guangdong | O177:H45 | α1 | 2820 | KT591270 |
| EP354 | healthy carrier | 2014 | Guangdong | O139:H19 | η2 | 2847 | KT591291 |
| EP355 | healthy carrier | 2014 | Guangdong | O107:H31 | ζ3 | 2817 | KT591326 |
| EP356 | healthy carrier | 2014 | Guangdong | O136:H21 | θ | 2808 | KT591241 |
| EP357 | healthy carrier | 2014 | Guangdong | O145:H34 | ι1 | 2814 | KT591298 |
| EP358 | healthy carrier | 2014 | Guangdong | O138:H48 | η2 | 2847 | KT591290 |
| EP359 | healthy carrier | 2014 | Guangdong | O167:NT | ζ3 | 2817 | KT591325 |
| EP360 | healthy carrier | 2014 | Guangdong | O85:NT | ζ3 | 2817 | KT591324 |
| EP361 | healthy carrier | 2014 | Guangdong | O171:H19 | ε2 | 2847 | KT591275 |
| EP362 | healthy carrier | 2014 | Guangdong | O139:H19 | η2 | 2847 | KT591289 |
| EP363 | healthy carrier | 2014 | Guangdong | O129:H11 | ο | 2820 | KT591318 |
| EP364 | healthy carrier | 2014 | Guangdong | O13:H11 | ο | 2820 | KT591317 |

^a^ NT, nontypable
